# *QuickStats:* Percentage* of All Emergency Department (ED) Visits^†^ Made by Patients with Diagnosed Depression,^§^ by Sex and Age Group — National Hospital Ambulatory Medical Care Survey, United States, 2016

**DOI:** 10.15585/mmwr.mm6746a6

**Published:** 2018-11-23

**Figure:**
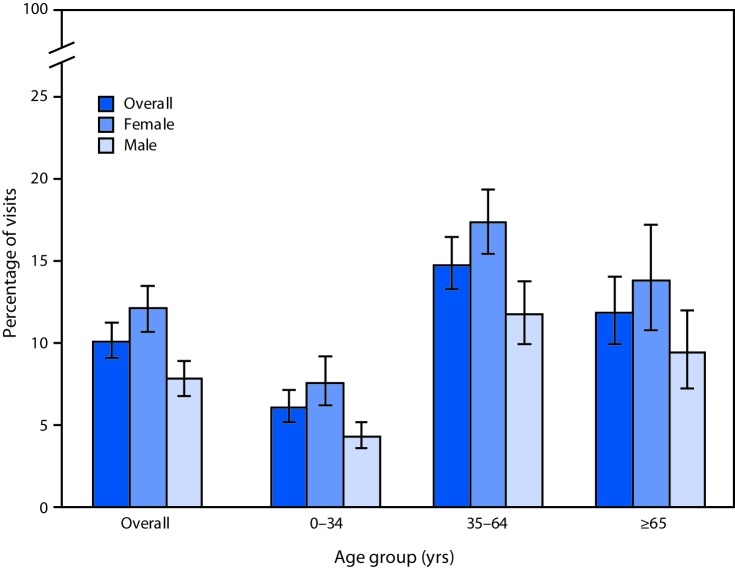
During 2016, 10.1% of all ED visits in the United States were made by patients with depression documented in their medical record. By age, the highest percentage of ED visits by patients with depression was for visits by patients aged 35–64 years (14.8%), compared with 6.1% for visits by patients aged 0–34 years and 11.9% for patients aged ≥65 years. A higher percentage of visits to the ED were made by females with depression (12.1%) compared with males with depression (7.8%). This same pattern was present for all three age groups.

**Source:** National Center for Health Statistics, National Hospital Ambulatory Medical Care Survey, 2016.

